# Induction and repetitive embryogenesis of *Ocotea porosa*

**DOI:** 10.1186/1753-6561-5-S7-P148

**Published:** 2011-09-13

**Authors:** Marguerite Quoirin, Luciana Pelegrini, Luciana Ribas

**Affiliations:** 1 Universidade Federal do Paraná, Brazil; 2 UFPRm Brazil

## Introduction

*Ocotea porosa* (Liberato Barroso) known as “imbuia” belongs to the Lauraceae family and is native to the Mixed Ombrophilous Forest (Araucaria Forest) where it was heavily exploited due to the high quality and worldwide value of his hardwood that is exported in large quantities for luxury furnishing manufactures [1]. The sexual propagation of *O. porosa* at its natural occurrence area is difficult, due to the strong tegumentary dormancy and its irregular germination. Moreover, the seed viability is short by virtue of being a recalcitrant species, showing high level of humidity [1]. Another limiting factor for its vegetative propagation is the low response of cuttings to the induction of adventitious roots (4%) [2]. The aim of this study was to establish repetitive cycles of secondary somatic embryogenesis from *O. porosa* embryonic axes.

## Material and methods

Zygotic embryonic axes from immature seeds were used as explants. The seed disinfestation was performed in a laminar flow chamber, through immersion in ethanol 70% (v/v) for 5 min, followed by 20 min in NaOCl 4% (v/v) supplemented with 0,1% of Tween^®^ 20. After that, the seeds were rinsed five times with sterile water. The WPM [3] culture medium was used in all stages. Embryonic axes were inoculated on culture medium supplemented with sucrose (20 g L^-1^), activated charcoal (1.5 g L^-1^), agar Vetec® (4 g L^-1^) and2,4-D (200 µM) combined or not with hydrolyzed caseinor glutamine (0.5 or 1 g L^-1^) during 90 days. The somatic embryos obtained in the induction phase were multiplied on medium with sucrose (20 g L^-1^), agar Vetec^®^ (3.5 g L^-1^)and 2,4-D (22.62 µM) combined with 2-iP (2.46 µM) for 90 days followed by transfer to culture medium with 0.5 g L^-1^ of hydrolyzed casein combined with 0.5 g L^-1^ glutamine or 1 g L^-1^ hydrolyzed casein or 1 g L^-1^ glutamine for 30 days. The maturation of somatic embryos was tested in WPM culture medium containing sucrose (20 g L^-1^), agar Vetec® (3.5 g L^-1^)and polyethylene glycol (PEG 6000) (control, 3.5 and 7%) during 30 days. The experiment was repeated twice. All the cultures were maintained in the dark at 27±2°C (day) and 18±2°C (night).

## Results and discussion

Patterns of direct and indirect induction of somatic embryos were observed with low frequency in culture medium containing 200 µM 2,4-D alone or combined with 1 g L^-1^ hydrolyzed casein or glutamine. The mean percentage of calli with somatic embryos varied between 4.2% and 8.3% for primary somatic embryogenesis without differences between treatments (after 90 days). In the present study, the maximum percentage of somatic embryos induction was 8.3%. Similar results were found with *O. odorifera* that presented a mean percentage of 6.5% [4]. On the culture medium containing 200 µM 2,4-D, the percentage of calli with somatic embryos was 6.3% and the formation of one globular embryo was visible. When 200 µM 2,4-D was combined with 1 g L ^-1^ hydrolyzed casein this percentage was 8.3% and two globular somatic embryos were developed per callus. However, in the medium containing 200 µM 2,4-D and 1 g L^-1^ glutamine, after 90 days the percentage was smaller (4.2%) and two globular and one cordiform shape embryos were observed. After three months, the primary somatic embryos subculturedin culture media without plant growth regulators with or without activated charcoal did not progress to torpedo and cotyledonary stages. In repetitive embryogenesis the combination of 0.5 g L^-1^ of hydrolyzed casein and glutamine promoted an average of 46.6 new globular embryos per callus and 75% of callus presented embryogenic mass com pro-embryos. The subculture in media containing 1 g L^-1^ hydrolyzed casein promoted an average formation of 58 globular embryos, 2 cordiform, 2.5 torpedo and 1 cotiledonary per callus at the end of every subculture and 72.5% of explants formed mass containing pro-embryos. When 1 g L^-1^ glutamine was added into the media, 57.5% of the mass presented pro-embryos and the induction of globular somatic embryos was lower (23.3 globular embryos per explant). During maturation phase, the somatic embryos develop from initial to late stages. However, in the present conditions of maturation, the percentage of embryos developing to cordiform, torpedo and cotyledonary ontogenetic stages was low, revealing an asynchronous development. In both treatments (3.5 and 7% PEG) as well as in the control, the first response was the formation of pro-embryogenic masses in 100% of calluses. In the control and in the medium supplemented with 3.5% PEG, an average of 32.7 and 27 new globular embryos were formed per explant, respectively. When PEG concentration in culture medium was 7%, the formation of new globular embryos decreased (12.7), but the development of cordiform (1.5), torpedo (2) and cotyledonary embryos (2) was observed.

## References

[B1] Carvalho PER Espécies arbóreas Brazileiras 2003 1 Colombo: Embrapa Florestas 1040

[B2] Inoue MT Putton V Macropropagação de 12 espécies arbóreas da Floresta Ombrófila Mista Floresta 2007 37 55 61

[B3] Lloyd G McCown B Commercially feasible micropropagation of mountain laurel, *Kalmia latifolia*, by use of shoot tip culture Proceed Int Plant Propagation Soc 1980 30 421 427

[B4] Santa-Catarina C Maciel SC Pedrotti EL Germinação in *vitro e* embriogênese somática a partir de embriões imaturos de canela sassafrás (*Ocotea odorifera* Mez) Revista Brazileira de Botanica 2001 24 501 510

